# Green Perovskite Distributed Feedback Lasers

**DOI:** 10.1038/s41598-017-11569-3

**Published:** 2017-09-15

**Authors:** J. R. Harwell, G. L. Whitworth, G. A. Turnbull, I. D. W. Samuel

**Affiliations:** Organic Semiconductor Centre, School of Physics and Astronomy, University of St Andrews, North Haugh, St Andrews, KY16 9SS United Kingdom

## Abstract

A visible perovskite distributed feedback laser is fabricated for the first time. Through the use of nanocrystal pinning, highly luminescent methylammonium lead bromide films are used to produce stable lasers emitting at 550 nm, with a low threshold of 6 µJcm^−2^. The lasers were able to support multiple polarisations, and could be switched between transverse magnetic and transverse electric mode operation through simple tuning of the distributed feedback grating period.

## Introduction

Advances in semiconductor laser materials have opened up many applications from data storage to sensing, spectroscopy, displays and lighting. In the visible region of the electromagnetic spectrum, commercial indium gallium nitride diode lasers now cover discrete wavelengths ranging from 375 nm to 525 nm while aluminium gallium indium phosphide lasers generate red light from 635 nm, but there remains a “green gap” in the spectrum of available wavelengths. Organic semiconductor lasers can span the whole visible spectrum and so can potentially fill this gap. They can readily be pumped by laser diodes and light-emitting diodes, and can be made via solution processing on nanoimprinted distributed feedback resonators for simple integration on a variety of substrates. However the accumulation of triplet excitons mean that organic lasers are currently limited to short pulses, and their photostability needs improvement. While enhancements in material stability and laser performance continue to make progress, alternative solution processable gain materials such as organometal halide perovskites offer a promising new route to overcome these limitations.

Since their discovery as efficient solar cell materials in 2009^1^, methylammonium lead halide perovskites and their variants have proven to be a highly disruptive discovery in the field of thin film photovoltaics. Their combination of high absorption coefficients, excellent charge mobilities^2^ and diffusion lengths^3^, as well as low cost solution processable starting materials make them extremely promising, allowing them to reach power conversion efficiencies of 22.1% as of 2016^4^. One of the key features of perovskites which gives them such high performance is that perovskite solar cells have particularly high open circuit voltages, which can be attributed to a high electroluminescence external quantum efficiency (EQE) of the material^5,6^. Indeed, much of the work on maximising the efficiency of perovskite solar cells now revolves around optimising the EQE of the material, and the best solar cells now also double as light-emitting diodes with surprisingly high efficiency^6^. This high luminescence efficiency has made perovskites an attractive prospect as a new class of light-emitting material for thin film light-emitting diodes and lasers. Recently a near infrared perovskite light-emitting diode (PerLED) has been reported with an EQE of 11.7%^7^, and several optically pumped perovskite lasers from perovskite nanowires^8^ and microdisks^9^ have also recently been reported. Distributed feedback (DFB) perovskite lasers, which allow much greater control over the laser beam shape and wavelength have also recently been reported in the literature^10,11^. These exhibit good thresholds and stability^12^, however they have thus far been confined to emission in the infrared.

In this report we demonstrate the first visible perovskite distributed feedback laser. By using nanocrystal pinning to fabricate highly luminescent methylammonium lead bromide perovskite films with low scattering losses, we produce green lasers with low threshold. We also perform an in-depth study on the properties of perovskites lasers, and we observe that the isotropic nature of the emission process in the perovskite thin films allows strong optical gain in both TE and TM optical modes, and allows the polarisation of the surface-emitted output beam to be switched between azimuthal and radial polarisations simply by fine tuning the feedback grating period. Finally, we show that perovskite lasers can have good stability due to their high resistance to oxidation in atmosphere. While perovskites are notorious for being water sensitive, we show that a hydrophobic fluorinated polymer cladding layer on top of the lasers allowed them to remain operational for months at a time. With this simple encapsulation, we show that the lasers can be operated continuously for long periods of time with no measurable degradation even in ambient conditions.

## Results and Discussion

**Figure 1:**
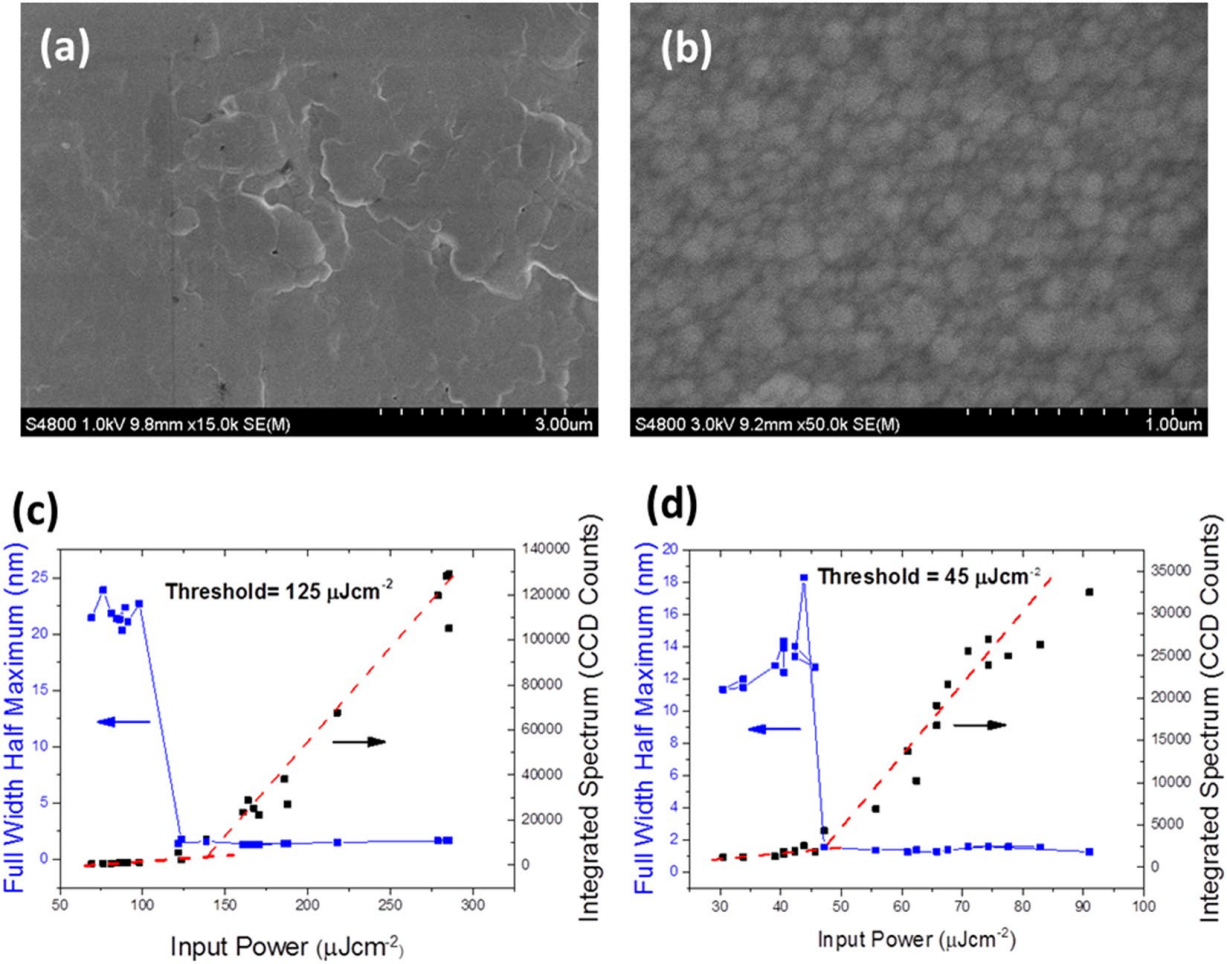
Scanning electron microscopy images of perovskite formed by the PbAc route (**a**), and the nanocrystal pinning route (**b**). (**c**) and (**d**) show the ASE thresholds for the PbAc route and the A-NCP route respectively.

A good thin film gain medium for distributed feedback lasing must simultaneously exhibit a high photoluminescence quantum yield (PLQY) in order to get maximal gain, and low scattering so as to minimise waveguide losses. Therefore the optimal film for a perovskite laser would have as low roughness as possible, whilst still maintaining a high PLQY. A major issue with perovskites is that their properties are extremely sensitive to their fabrication conditions, and the same material made by different routes can result in very different properties. For example, a minor change in the environment can have a strong adverse effect on film quality. As such it is common to make perovskite films which have high PLQY but poor roughness, or vice versa, but simultaneously optimising both surface roughness and PLQY is not a straightforward task. In order to find the most suitable fabrication route for perovskite lasers, we compared the two main methods of fabricating solution processed perovskites – the simple one-step method, and the nanocrystal pinning route. For the one step method, the lead acetate (PbAc) route described by W Zhang *et al*. was used^13,14^. This route is known to give perovskite films of high optical quality and low roughness, however the PLQY of the films we made with this method was lower than the resolution of our measuring setup (i.e. less than 1% at 442 nm continuous wave excitation). An SEM image of a typical film from this route is shown in figure 1(a). The root mean square (r.m.s) surface roughness was also measured via atomic force microscopy (AFM) to be 15 nm over a 5 × 5 µm area (see Figure S7(a)), which is in reasonable agreement with the value of 12 nm over a 15 × 15 µm area Zhang demonstrated when the technique was first reported.

Of the various approaches to depositing perovskites via nanocrystal pinning, an adapted version of the assisted nanocrystal pinning (A-NCP) technique, described by Tae Woo Lee *et al*.^15^ produced the best balance between high PLQY and good film quality. This involved nanocrystal pinning of the sample using an antisolvent doped with 2,2′,2″-(1,3,5-Benzinetriyl)-tris(1-phenyl-1-H-benzimidazole) (TPBi), which was found to improve PLQY by forcing the creation of smaller, higher quality crystals. To optimise this procedure for lasing we explored a range of antisolvents including toluene, chlorobenzene, chloroform, dichloromethane and diethyl ether at a range of TPBi concentrations from 0 to 20 mgml^−1^ and found that an antisolvent of dichloromethane (DCM) with a 5 mgml^−1^ concentration of TPBi gave the most luminescent and highest quality films. Compared to the PbAc route, this method gives a much higher, albeit still modest PLQY of 5% when measured under 442 nm continuous wave (CW) laser excitation. However the quality of films produced by this method was found to be much more variable, and the films visually appear to have more light scattering than films from the lead acetate route. An SEM image of a typical film is shown in figure 1(b). Despite higher scattering and more crystal grain boundaries, AFM images of the samples showed a remarkably low surface roughness of 1.3 nm over a 5 × 5 µm area (see Figure S7(b)), which we attribute to the films being completely pinhole-free.

To investigate which was likely to be the better method for laser fabrication, amplified spontaneous emission (ASE) measurements were made for each type of film. The results are shown in figure 1(c) and (d). For the sample prepared by the PbAc route, the emission output increases steeply and the spectrum line width collapses to approximately 2 nm  for pump energy densities greater than 125 µJcm^−2^, indicating that this is the ASE threshold. Films made from the A-NCP method exhibit similar behaviour, but at a lower threshold energy density of 45 µJcm^−2^. Therefore films made via the A-NCP route have an ASE threshold which is nearly a factor of three lower than films produced from the PbAc route. Given that the PLQY of the films produced from the A-NCP route is at least an order of magnitude larger than those from the PbAc route, one might expect the ASE threshold of the A-NCP perovskite to be even lower^16^. We therefore explored whether this discrepancy was due to differences in waveguide losses. Comparing scanning electron microscopy images of the films from both routes (see figure 1), the PbAc route produces films with a smooth landscape with crystal edges very poorly defined. This is expected to give low scattering losses despite the presence of pinholes increasing the r.m.s roughness. The A-NCP route on the other hand shows a dense network of ~100–300 nm crystals with clear boundaries between crystals. Given that it is made up of an array of particles of the same length scale as the waveguided light, it is indeed remarkable that the films made by the A-NCP route support ASE at all, as one would expect scattering off the crystal boundaries to introduce large losses to the system. The waveguide loss was measured and found to be 8 cm^−1^ for films from the A-NCP route, and 2 cm^−1^ for films from the PbAc route. Thus we find that, although the more defined crystal boundaries in the A-NCP route do introduce significantly more waveguiding losses due to scattering, the increased loss is more than offset by the higher PLQY.

**Figure 2:**
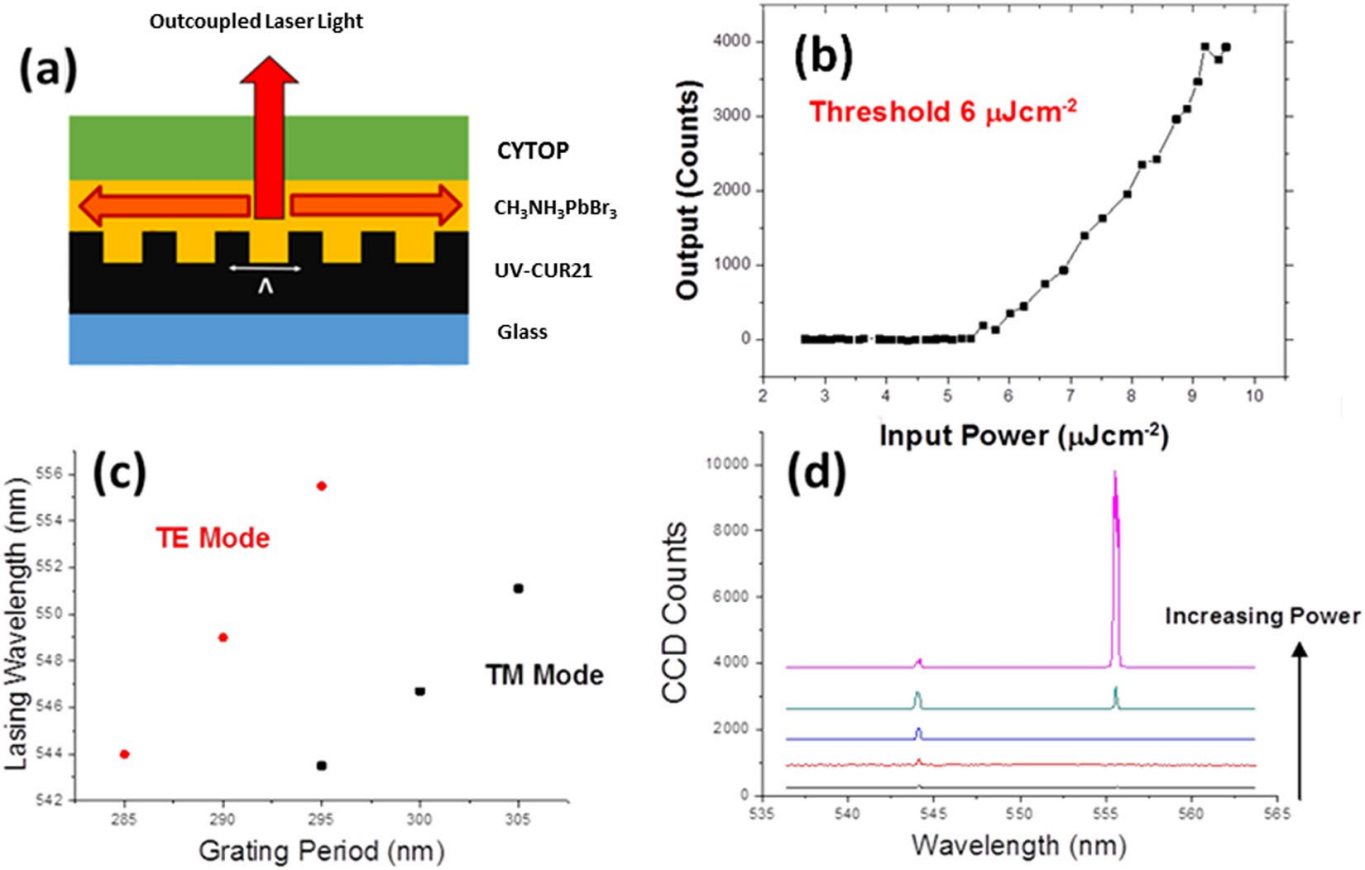
**(a)** The structure of the final laser design. (**b**) The threshold measurement for the lowest threshold laser, showing a marked change in slope at the threshold energy density of 6 μJcm^−﻿2^﻿. (**c**) The lasing wavelength vs grating period for the TE and TM modes of a particular grating array. (**d**) The emission spectrum of a 295 nm grating for 4 different pump powers between 15 and 30 µJcm^−2^.

Because of its lower ASE threshold, the A-NCP route was chosen for making a distributed feedback laser. The target laser structure is shown in figure 2(a) and consists of a perovskite film spin coated directly onto a two-dimensional grating structure. The grating period was tuned to provide second order distributed feedback for light at the expected lasing wavelength of 550 nm. A standard spin coating method gave a perovskite film of ~140 nm thickness (measured by stylus profilometry) which, based on literature measurements of the perovskite refractive index^17^, gives an effective refractive index for the TE waveguide mode of 1.91, so a grating period of 290 nm was expected to provide second order distributed feedback for the laser. To make the laser, an array of two dimensional feedback gratings of periods between 250 and 425 nm in 5 nm steps was made by ultraviolet nanoimprint lithography (UV-NIL). Each grating was a 2 mm × 2 mm square, spaced to be 2 mm apart. The perovskite layer was deposited on the gratings by the A-NCP method, and transverse SEM images show that the perovskite conforms well to the grating structure (see Figure S6). The finished device was then encapsulated by spin coating a layer of CYTOP^TM^, a commercial amorphous fluoropolymer, on top of the perovskite. CYTOP^TM^ provides a weak barrier to oxygen but an excellent barrier to water, making it an ideal encapsulant for this system, as water is the main degradation catalyst for methylammonium lead bromide^18,19^. Oxygen on the other hand is not known to be detrimental to perovskites, and has even been shown to assist photoluminescence and solar cell performance^20^. We briefly studied the effects of oxygen on the laser action by measuring the laser inside a vacuum chamber; however we did not observe any significant shifts in lasing threshold between vacuum and atmosphere.

The complete device had a structure as shown in figure 2(a). When the sample was excited with 355 nm, 1 ns pulsed laser light, laser action was observed with a minimum threshold pump density of 6 µJcm^−2^ on the Λ = 300 nm grating – see figure 2(b). Above threshold the width of the spectrum was less than 1 nm, limited by the spectral resolution of the spectrograph. The laser wavelength could be tuned to a small degree by changing the grating period as shown in figure 2(c). For grating periods between 305 nm and 295 nm the laser wavelength changed linearly from 552 nm to 543 nm, but when the grating period was reduced further the emission wavelength hopped to 555 nm before continuing the linear trend. In some, but not all devices the Λ = 295 nm period grating supported two spectrally separated laser modes - the spectrum of an example laser is shown in figure 2(d). The laser initially turned on at 544 nm above a threshold pump density of 15 µJcm^−2^, with a second mode at 555 nm appearing with stronger pumping. This mode grew much faster with pump power and at 30 µJcm^−2^ it dominated the spectrum.

**Figure 3:**
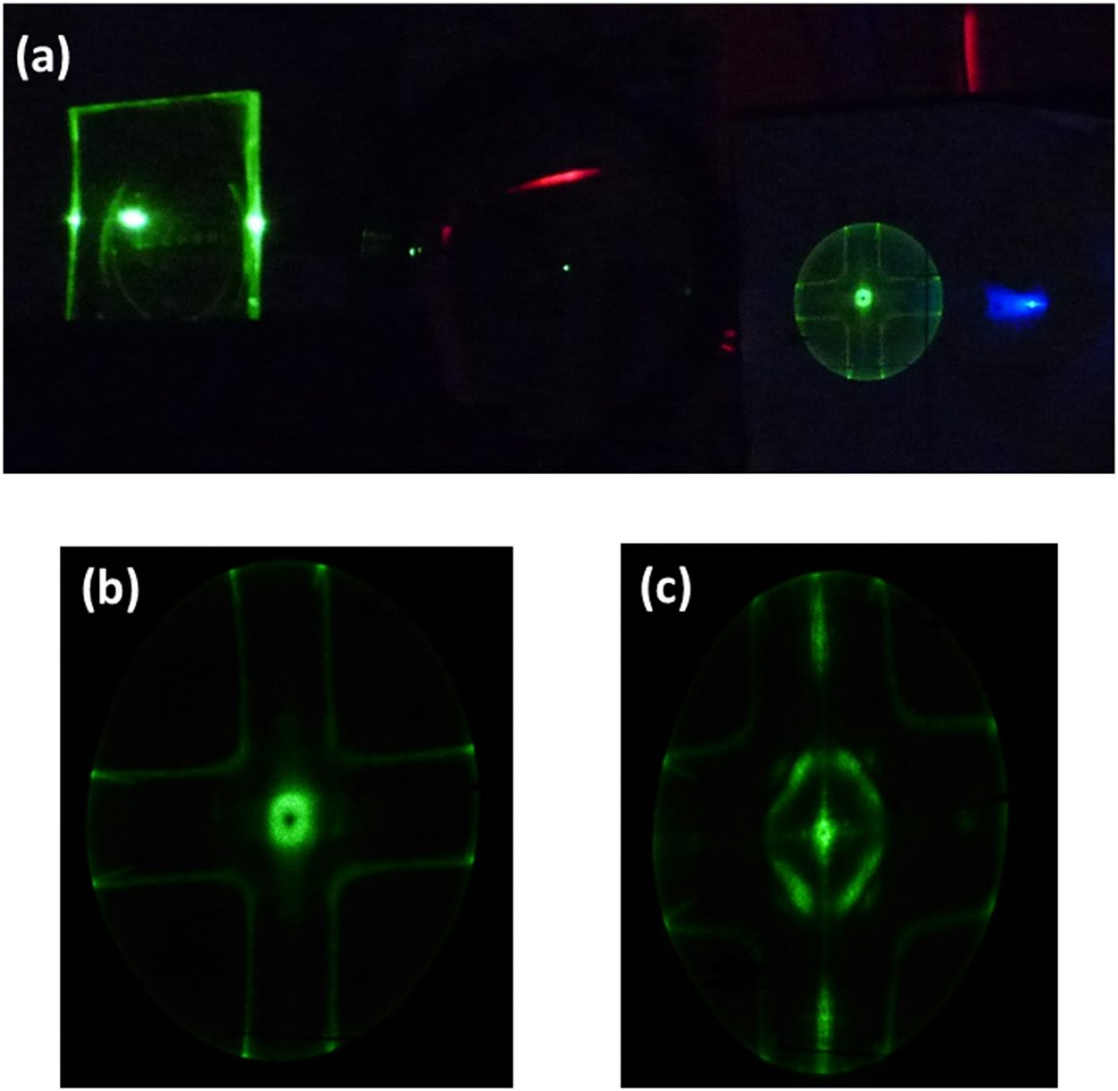
(**a**) Shows a perovskite laser in operation, with the output projected onto a white screen. (**b**) Is a close up photograph of the TM mode from a 300 nm grating, while (**c**) shows the TE mode from a 290 nm grating.

A photograph of the laser under normal operation is shown in figure 3(a). The output is dominated by an annular transverse mode which is attributed to a 2D Bloch resonance supported by the two orthogonal grating vectors of the structure. Other scattered light visible in the picture is caused by waveguided light being outcoupled by adjacent gratings on the substrate – the fact that these are visible further indicates the low propagation losses in this material. Figure 3(b) and (c) show close ups of the output beams from gratings of 300 nm and 290 nm periods respectively. Note that the shape of the output beam is significantly different between the two grating periods due to mode hopping between the different periods.

**Figure 4:**
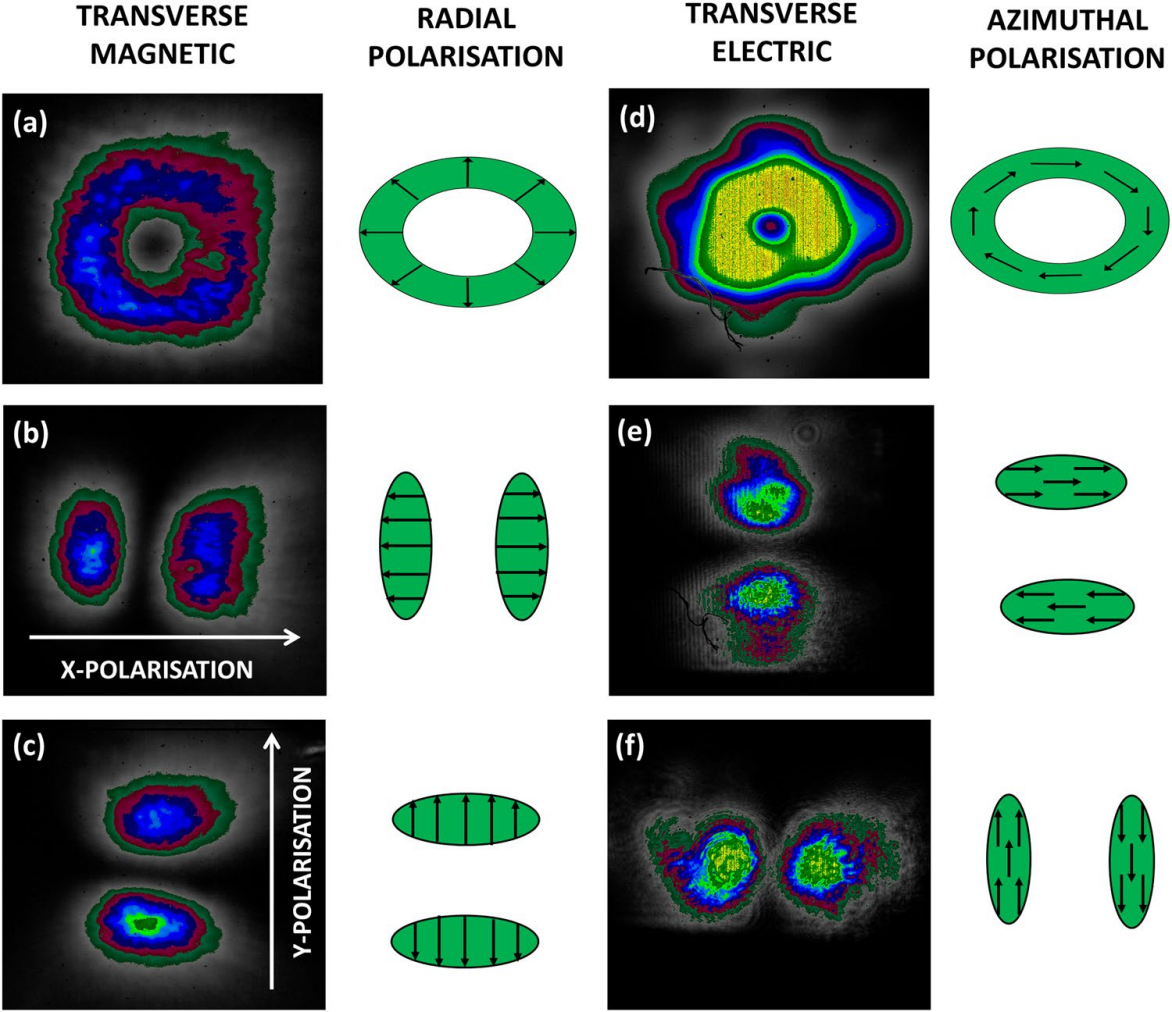
Beam profiles of perovskite laser output when viewed through analysers transmitting different polarisations – TM mode (**a**) unpolarised, (**b**) horizontally polarised, (**c**) vertically polarised, and TE Mode (**d**) unpolarised, (**e**) horizontally polarised, (**f**) vertically polarised.

To analyse the properties of the two observed modes further, measurements were taken of the output beams using a laser beam profiler (figure 4). As expected, we observe that the beam profile is an annulus for both modes (figure 4a,d). When the beams are viewed through a linear polariser a double lobed (TEM_10_ like) beam is observed (figure 4b,c,e,f), with the orientation of the double lobed beam being rotated by 90 degrees between the two modes. Using this information, we find that the surface emitted beam from the 290 nm grating is azimuthally polarised, as has been previously found for the surface emission from the transverse electric (TE) mode in polymer lasers^21^. The output from the 300 nm grating on the other hand is radially polarised, indicating that it originates from a transverse magnetic (TM) mode. The TM mode is not normally visible in polymer lasers because the strongly anisotropic nature of organic polymer films(long polymer chains strongly oriented to lie in the plane of the film) means that the TM mode experiences a very different effective refractive index^22^. Therefore, since most distributed feedback gratings are optimised for TE modes, the resonance condition for the TM mode is rarely met. The CH_3_NH_3_PbBr_3_ films on the other hand have a simple cubic perovskite structure with similar indices in the x, y and z directions. This means that the TE and TM modes will see very little difference in effective refractive index, and so small changes in grating period or effective refractive index can cause the laser to hop between TE and TM polarisations. Tuning the laser such that small changes in the local environment causes a switch in polarisation state could have sensing applications, and the ability to produce radially polarised beams are also of interest since it has been hypothesised that a radially polarised beam can be focussed to a spot size smaller than the diffraction limit^23^. Beating the diffraction limit with these particular lasers however is unlikely because the M^2^ beam quality factor was measured for the TE and TM lasers and found to have values of 26 for TE and 36 for TM (see Figure S2). The lowest possible M^2^ value for an annular beam is 2, for a Laguerre-Gaussian LG_10_ beam. The higher M^2^ observed for these beams is due to the wider minimum in intensity on axis of the surface–emitted output beam from the DFB cavity.

The double mode behaviour on the 295 nm grating is caused by competition between the TE and TM modes which, due to their small difference in effective refractive index, are both within the perovskite’s gain region at this grating period. The TM mode has the lowest threshold so it appears first, but at higher pump density the TE mode appears and starts to compete for gain. Of particular interest is that the TE mode grows much faster with pump intensity than the TM, eventually dominating the spectrum as shown earlier on Fig. 2(d). This unusual behaviour would suggest that the TM mode has a lower surface emission loss than the TE mode. It is worth noting that only half of the fabricated laser devices demonstrated this double peak behaviour on the 295 nm grating (instead they would lase on just one of the two modes), but all devices would hop between the TE and TM modes when the grating period was changed sufficiently. We attribute this to small variations in the thickness of the perovskite layer between batches. The double mode will only appear when the thickness is such that one of the gratings has the resonance condition for both modes in a strong part of the gain profile. This is likely to be a narrow window, and since the grating periods change in 5 nm steps it would be possible for one to miss this point at certain film thicknesses.

To confirm further that our assignments of the various modes as TE and TM were accurate, we performed finite difference time domain (FDTD) simulations using refractive index data reported by Alias *et al*.^18^ to attempt to reproduce the observed lasing modes on the dual mode operation in the Λ = 295 nm grating (543.5 and 556.0 nm). The situation is complicated somewhat by the fact that the refractive index of CH_3_NH_3_PbBr_3_ varies strongly with wavelength in this region. Using the refractive index values for CYTOP (1.33), UVCur-21 (1.53), and CH_3_NH_3_PbBr_3_ at 556 nm (2.165) gives an effective refractive index for the TE mode of 1.884. This predicts a lasing wavelength of 556 nm which is exactly what we observe. Doing the same for the TM mode, whilst being careful to account for the dispersion in refractive index, gives a TM mode effective refractive index of 1.832. This predicts a lasing wavelength of 540 nm, which is 3 nm blue shifted compared to what we experimentally observe. We attribute this small discrepancy to our film having a slightly different refractive index dispersion to that of Alias *et al*. due to us using a different deposition route. However the important point is that the FDTD simulations predict that the shorter wavelength modes are TM and the longer modes are TE, which is in direct agreement with our conclusions from the polarisation experiments described earlier. The solution curves to the effective refractive index are shown in Figure S8.

A key challenge for solution processed distributed feedback lasers is to demonstrate stable lasing under high powers, or ultimately continuous wave (CW) operation. With organic semiconductors this is a significant challenge for two reasons - firstly long lived triplet excitons, which do not emit light, can accumulate in the material over long pulses. These introduce additional absorption losses in the cavity and can quench the light-emitting singlet excitons, thus terminating the lasing^24^. Secondly for high average intensities the material can be quickly degraded by the pump beam due to photo-oxidation and thermal degradation^24^. Perovskites are known to have weak exciton binding energies^25^, so triplet states should not significantly accumulate, and they are more resistant to degradation from thermal and photo-oxidation effects, making them a very attractive candidate for high average power operation.

Due to their known high resistance to oxidation, the perovskite lasers proved to be very stable in atmosphere, and so long as they were encapsulated with CYTOP they would remain operational even if left in ambient atmosphere with 50% humidity for long periods of time. Laser action was achieved from a perovskite sample after more than 30 days in ambient conditions, albeit at an increased threshold of 50 µJcm^−2^, and a perovskite laser could be studied continuously over an entire day with no noticeable degradation of the material. To assess this observed stability of the perovskite laser quantitatively, a perovskite laser was pumped at ~2x threshold with 20 Hz pulse rate and left to operate overnight. The lasing output versus time under these conditions was measured, and it was found that the output power remained constant over the entire test duration of more than 15 hours of continuous excitation (shown on Figure S5). This result underlines the good stability of perovskites as a laser gain medium and also leads to the conclusion that perovskites are an excellent candidate material for thin film continuous wave (CW) lasers, as they have the potential to overcome the degradation hurdle which is an important step on the road to CW operation.

In conclusion, we have demonstrated an all solution processed, visible emitting distributed feedback laser made from methylammonium lead bromide perovskite. We have shown that low thresholds of 6 µJcm^−2^ can be reached by careful selection of the fabrication route. Perovskite samples with significantly higher PLQY than those demonstrated in this work have recently been reported^26,27^, and so higher optical gain should be possible in the future. With further improvement in thin film fabrication lower scattering should also be attainable, and if both of these parameters could be optimised to their full extent then extremely low thresholds could be achieved in these materials. Perovskites as a laser material show good stability, making them good candidates for high repetition rates or even CW operation. Finally, we demonstrate that their cubic crystalline nature allows the films to support laser action in multiple polarisations for a given grating, including the possibility to generate radially polarised beams and the potential to switch polarisations with a small change in the resonator properties. This work demonstrates the potential of perovskites as a solution processed laser material, and shows that they could prove an excellent alternative to conventional organic compounds in situations where stability and simplicity of use is of primary concern.

## Materials and Methods

### Perovskite Solution Preparation and Deposition

For the preparation of perovskite thin films two deposition routes were used. The lead acetate (PbAc) route, and the assisted nanocrystal pinning (A-NCP) route. For the Pbac method, a precursor solution was made by dissolving a 175.6 mg of lead acetate trihydrate (Sigma Aldrich) and 155 mg methylammonium bromide (Dyesol) in 1 ml of Dimethyl Formamide to make a 1:3 molar ratio solution. The solution was dissolved completely by 5 minutes of shaking without heating, and then 3 µl of Hypophosphorous acid was added, and the solution was shaken again for a further 5 minutes. For ASE measurements, glass substrates were cleaned by sonication in acetone and IPA, and then by plasma ashing for 3 minutes. The solution was spin coated at 2000 RPM for 60 s, and then left at room temperature for 5 minutes before annealing at 100 °C for a further 5 minutes.

For the A-NCP method, a precursor solution lead bromide and methylammonium bromide was dissolved in dimethyl sulfoxide (DMSO) in a 1:1.05 ratio to make a 0.6 M solution. Total weights were 220.2 mg PbBr_2_ and 70.54 mg methylammonium Bromide in 1 ml DMSO. The solution was dissolved overnight at 60 °C, and spin cast at 3000 RPM for 90 seconds. To perform the nanocrystal pinning step, a solution of 2,2′,2″-(1,3,5-Benzinetriyl)-tris(1-phenyl-1-H-benzimidazole) (TPBi) dissolved in Dichloromethane to a concentration of 5 mg ml^−1^ was dropped on the sample mid-way through the spinning process. The optimal time for dropping the solution was just before the DMSO evaporated. A test sample would always be spin coated first and the time when the film could be observed to become rough was noted. On subsequent samples the dichloromethane solution would be dropped onto the film 5–10 seconds before this point, which resulted in very smooth “Glassy” films. The films were then immediately annealed at 90 °C for 10 minutes. All processing for perovskites was done in a nitrogen filled glovebox with oxygen and water concentrations of <1 part per million.

### Fabrication of Lasers

Laser gratings were fabricated using an EVG 620 photomask aligner with custom nanoimprint lithography tooling. First a perfluoro-polyether daughter stamp was cast from a silicon master structure (cured by UV exposure) and subsequently used to nanoimprint into a grating structure on a spin-coated UVCur06 film under UV exposure. The resultant gratings were then plasma ashed at low power for 30 seconds to improve wettability, and the perovskite precursor was spin coated on top of the sample using the above A-NCP method. Finally, an encapsulating layer of CYTOP^TM^ was spin coated on top of the film at 2000 RPM for 60 seconds. To confirm that the perovskite was sufficiently penetrating the gaps in the laser grating, a transverse scanning electron microscopy (SEM) image of a laser sample without CYTOP encapsulant was taken using an FEI Scios^TM^ DualBeam system. First a focussed ion beam was used to cut a trench in the sample, before an SEM image was taken of the trench at a 52° angle of incidence. It was observed that, barring a small number of defects, the perovskite could completely fill the gaps in the grating (see Figure S6).

### ASE and Lasing measurements

In all lasing and amplified spontaneous emission (ASE) measurements, the sample was pumped at 355 nm with 1 ns pulses from a CryLaS frequency tripled Nd:YVO_4_ laser. For ASE, the beam was focussed into a stripe on the sample by a cylindrical lens, and the intensity controlled by a variable neutral density (ND) wheel. Waveguided light out of the edge of the film was measured with a fibre coupled Andor CCD spectrograph. The pump pulse energy was measured using a beam splitter to reflect a known portion of the beam onto a Coherent^tm^ calibrated photodiode. The beam area was measured through the use of a Coherent^tm^ Beammaster beam profiler.

To measure waveguide propagation loss, the sample and the coupling fibre were placed on a moving stage, and the stage was moved such that the pump stripe moved across the sample, forcing the waveguided ASE light to propagate through a varying length of the unpumped film. The measured spectrum at each data point was integrated and an exponential curve was fitted to a graph of integrated spectrum vs position.

For lasing measurements, the beam was focussed into a spot of area ~0.5 mm^2^ with a spherical lens, and the laser output was measured with the fibre. The sample was offset slightly from perpendicular to the incoming laser beam so that the pumping beam was not coupled into the fibre.

For stability measurements, the laser was left to be pumped overnight, and the output was measured at 60 second intervals using a CCD spectrograph. The output power was calculated by integrating the CCD counts across the lasing spectrum and then normalising to create a degradation curve.

The beam profile for the polarisation and M^2^ measurements was measured with a Coherent Beammaster beam profiler. M^2^ measurements were taken by focusing the laser output beam with a lens and measuring the beam radius versus displacement using the beam profiler on a moving stage. The M^2^ value was calculated by fitting the data to the beam radius equation $\,w={w}_{0}\sqrt{1+{(\frac{{M}^{2}\lambda z}{\pi {{w}_{0}}^{2}})}^{2}}$.

### Other Measurements

Photoluminescence quantum yield (PLQY) measurements were taken using a 442 nm continuous wave Helium-Cadmium laser with an output power of ~50 mW. The beam was incident on the sample in an integrating sphere. The laser and sample light was measured with a fibre coupled Andor CCD spectrograph, and the PLQY was calculated using the method described by Greenham *et al*.^28^. The spectral response of the CCD and the sphere was calibrated using a tungsten lamp of known spectrum. Thickness measurements were carried out using a Veeco Dektak 150 surface profilometer. Absorption measurements were taken using a CARY 300 UV-Vis Spectrometer. Scanning electron microscopy measurements for Fig. 1(a) and (b) were taken using a Hitachi S4800 scanning electron microscope.

### Data availability

All data underpinning this manuscript, as well as the results of preliminary experiments can be found at the following doi: http://dx.doi.org/10.17630/fa79eead-de1e-47ad-85d7-649b5e482f22.

## Electronic supplementary material

Supplementary Infomation
