# Supplementary material for: Green Perovskite Distributed Feedback Lasers
Source: Sci Rep. 2017 Sep 15;7:11727. doi: 10.1038/s41598-017-11569-3 (PMC5601482; doi:10.1038/s41598-017-11569-3)
Supplement: Supplementary file 1 — Supplementary Infomation [file 41598_2017_11569_MOESM1_ESM.pdf]

Green Perovskite Distributed Feedback Lasers

Supplementary Information

J.R.Harwell, G.L.Whitworth, G.A.Turnbull, I.D.W.Samuel[1]

Organic Semiconductor Centre, School of Physics and Astronomy, University of St Andrews, North Haugh, St Andrews, KY16 9SS

1. *Corresponding Author: Prof. Ifor Samuel, idws@st-andrews.ac.uk, (+44)1334 463114*

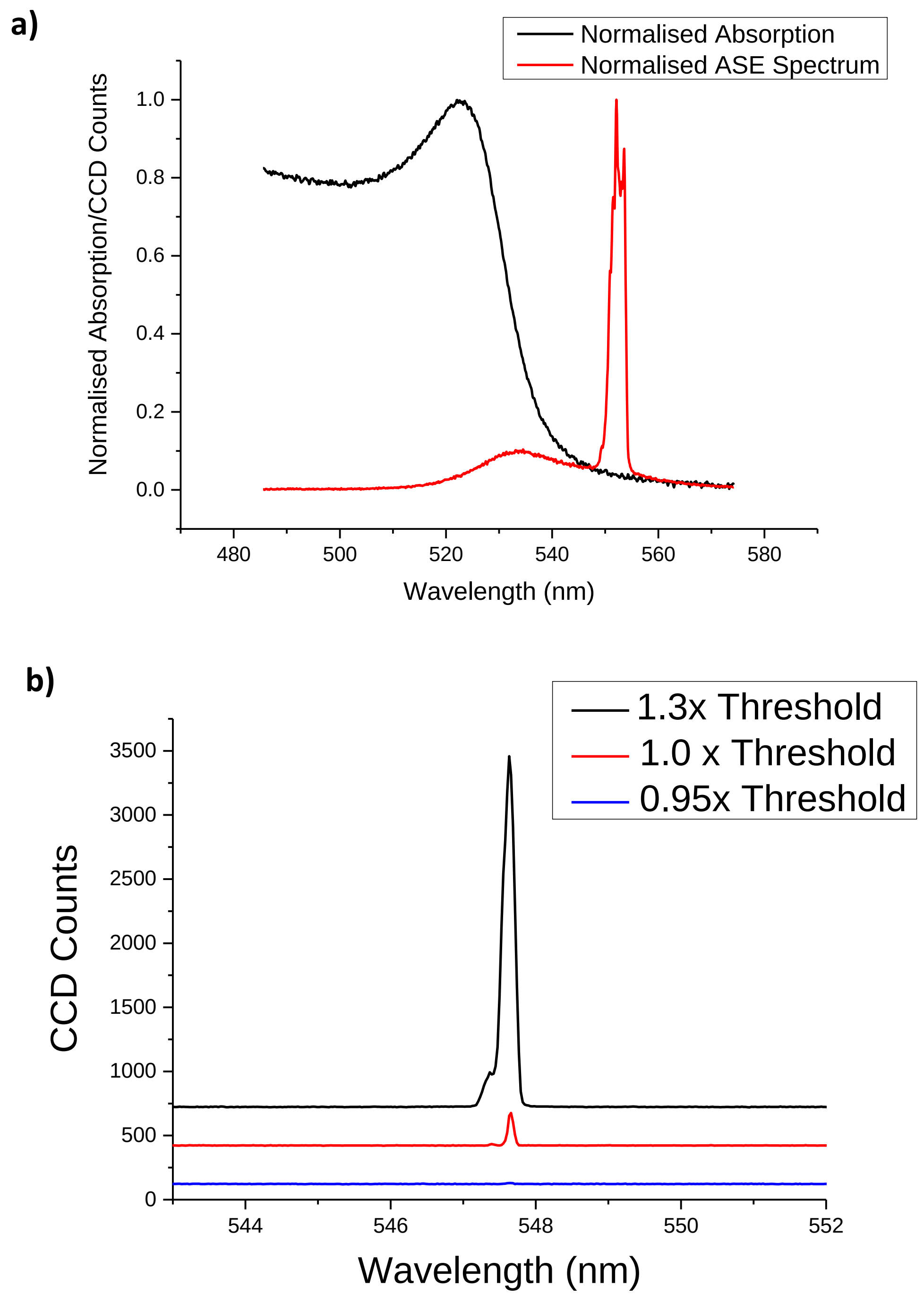

Figure. S1 – a)The absorption and ASE spectra of $CH_3NH_3PbBr_3$ and b) Single mode lasing spectrum of $CH_3NH_3PbBr_3$ on a Λ= 300 nm grating as threshold is crossed. Note that the ASE spectrum has negligible overlap with the absorption spectrum, meaning that self-absorption in the film does not contribute significantly to waveguiding losses

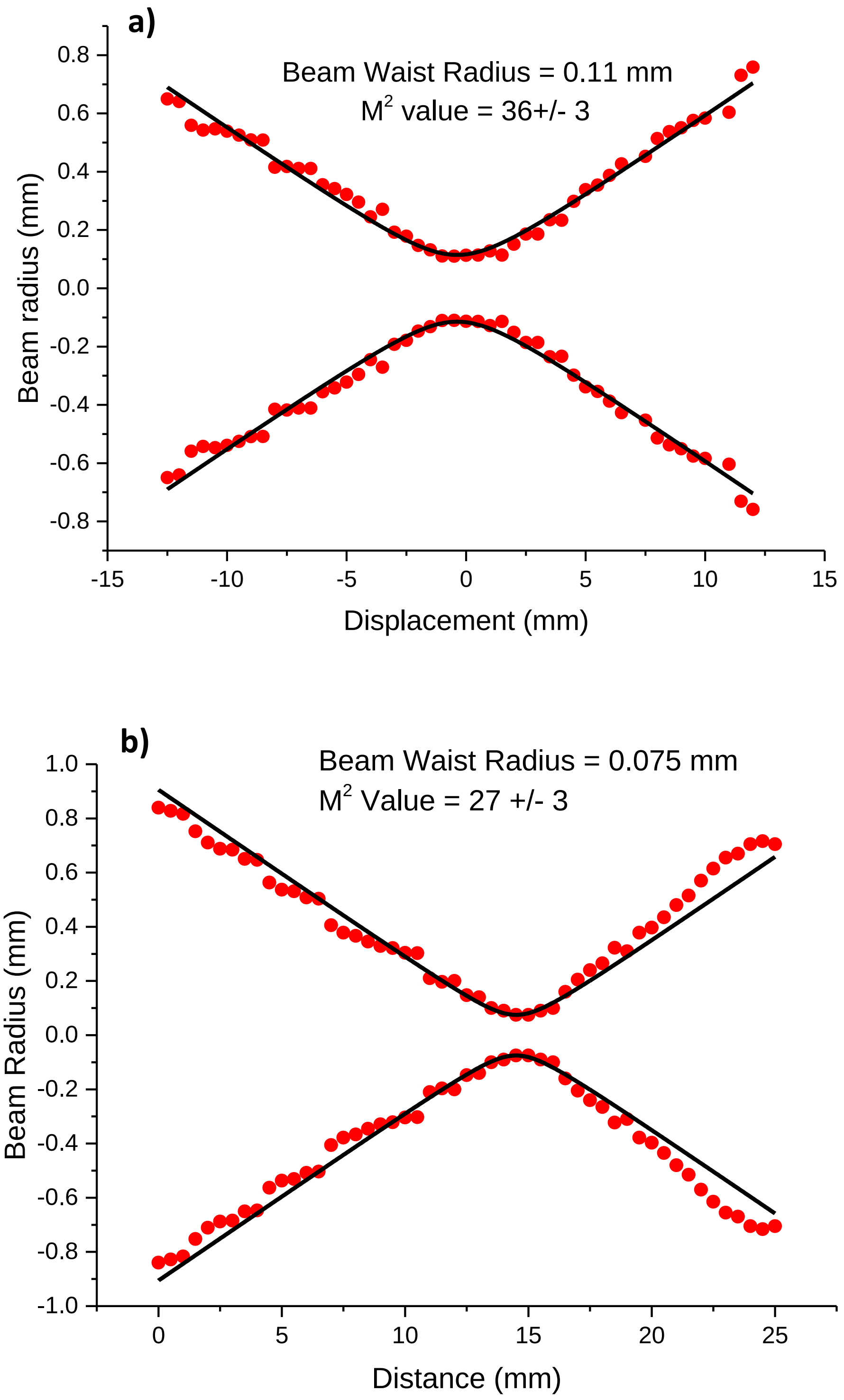

Figure. S2 – Calculation of the M^2 value of the a) TM mode and b) TE Mode from beam width vs displacement on a focussed beam

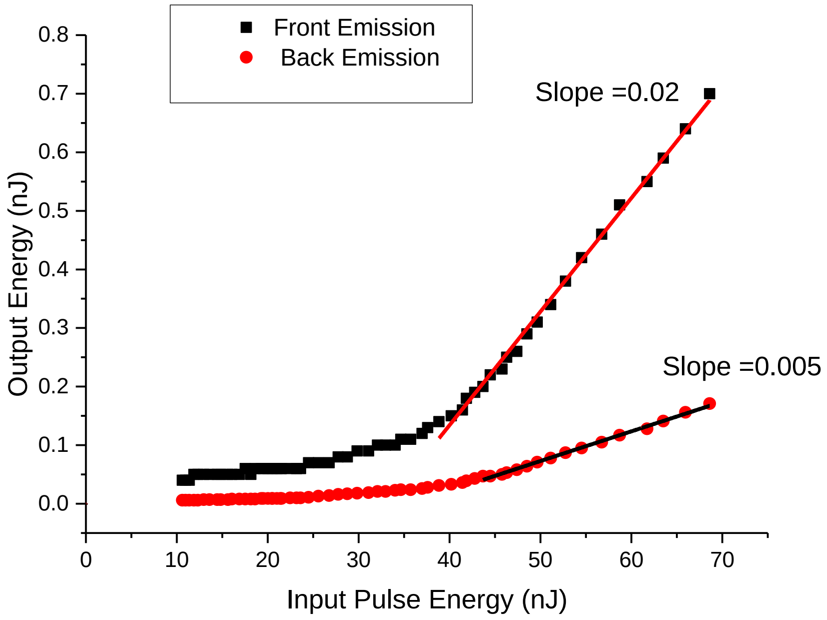

]

Figure. S3 – Calculation of laser slope efficiency

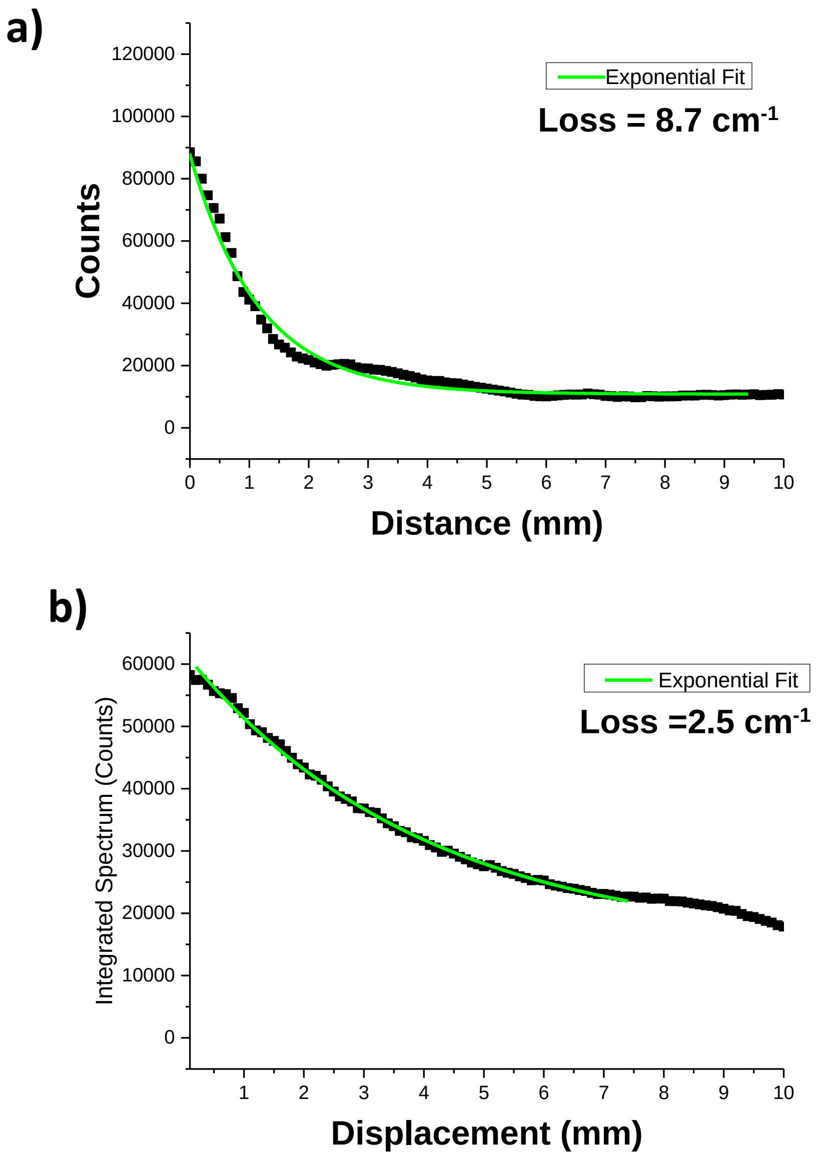

Figure. S4 - Loss Measurements for (a) PbAc route and the (b) nanocrystal pinning route

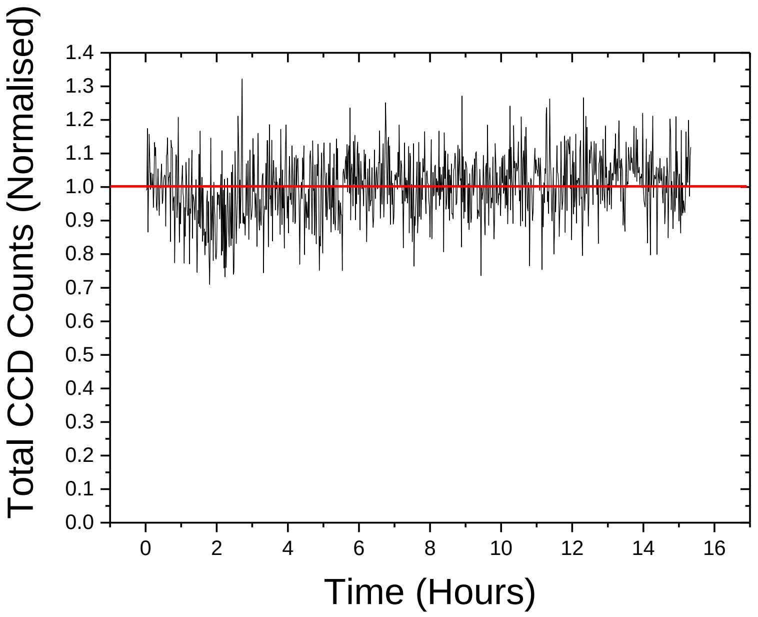

Figure. S5. Perovskite Laser output vs time at ~2x lasing threshold. Note that there is no observable degradation over 15 hours of continuous operation

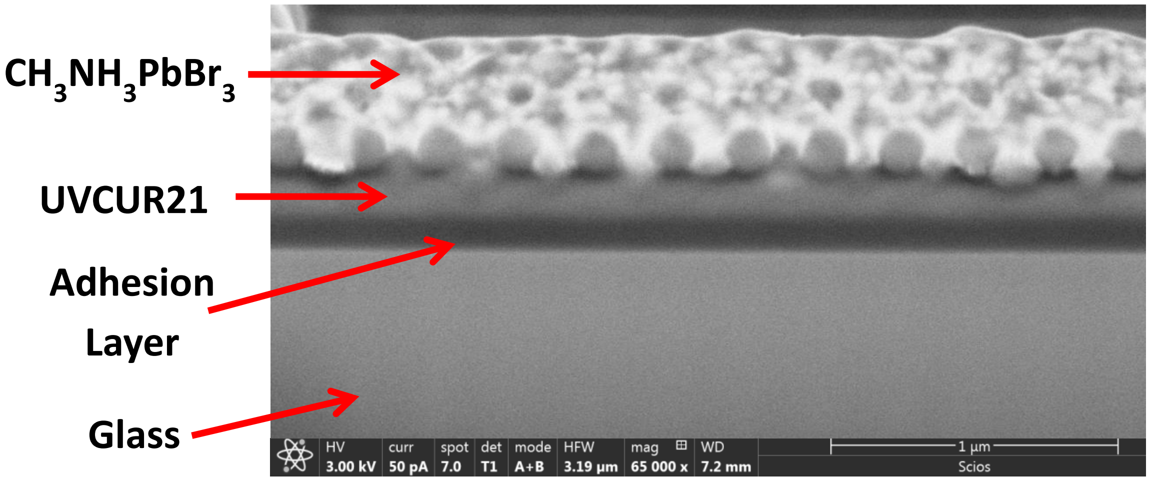

Figure S6 – Transverse SEM Image of perovskite deposited via the A-NCP route on a UVCUR21 grating.

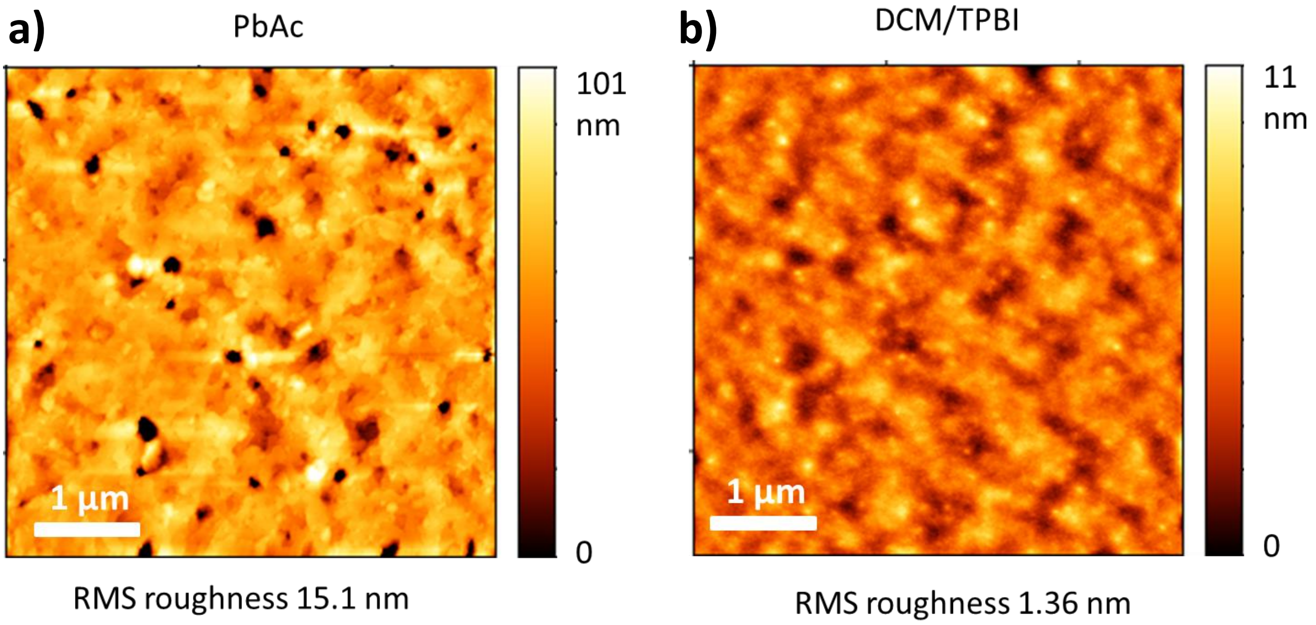

Figure S7 – Atomic force microscopy measurements of $CH_3NH_3PbBr_3$ films deposited on glass via (a) the pbac route and (b) the A-NCP route. R.M.S roughness is calculated over a 5x5 µm area.

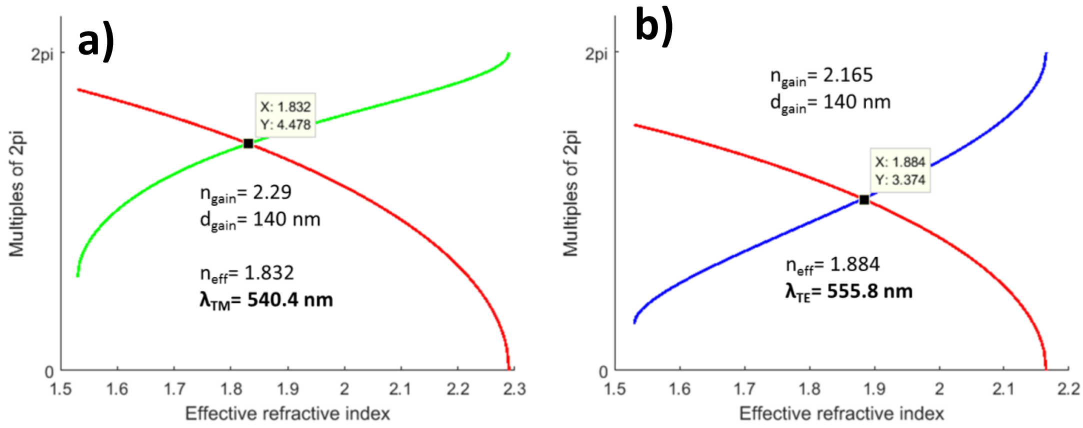

Figure S8 – FDTD simulation curves for (a) TM and (b) TE modes. The effective refractive index is defined as the point where the phase shift and geometric line cross. Calculations were performed assuming grating period of 295 nm, a perovskite layer thickness of 140 nm, and cladding layer refractive indices of 1.33 and 1.53 for CYTOP and the UVcur-21 grating respectively.
